# Association of apolipoprotein E gene polymorphisms with blood lipids and their interaction with dietary factors

**DOI:** 10.1186/s12944-018-0744-2

**Published:** 2018-04-30

**Authors:** Israa M. Shatwan, Kristian Hillert Winther, Basma Ellahi, Peter Elwood, Yoav Ben-Shlomo, Ian Givens, Margaret P. Rayman, Julie A. Lovegrove, Karani S. Vimaleswaran

**Affiliations:** 1 Hugh Sinclair Unit of Human Nutrition and Institute for Cardiovascular and Metabolic Research (ICMR), Department of Food and Nutritional Sciences, University of Reading, Whiteknights, PO Box 226, Reading, RG6 6AP UK; 2 Food and Nutrition Department, Faculty of Home Economics, King Abdulaziz University, Jeddah, Saudi Arabia; 3 Department of Endocrinology and Metabolism Odense, University Hospital Denmark, Aarhus, Denmark; 4 Faculty of Health and Social Care, University of Chester, Chester, CH1 1SL UK; 5 Department of Epidemiology, Statistics and Public Health, Cardiff University, University Hospital of Wales, Heath Park, Cardiff, CF14 4XW UK; 6 Population Health Sciences, University of Bristol, Bristol, BS8 2PS UK; 7 Institute for Food, Nutrition and Health, University of Reading, Earley Gate, Reading, RG6 6AR UK; 8 Department of Nutritional Sciences Faculty of Health and Medical Sciences, University of Surrey, Guildford, GU2 7XH UK

**Keywords:** *APOE* gene, Total cholesterol, LDL-C, PRECISE, Caerphilly prospective studies

## Abstract

**Background:**

Several candidate genes have been identified in relation to lipid metabolism, and among these, lipoprotein lipase (*LPL*) and apolipoprotein E (*APOE*) gene polymorphisms are major sources of genetically determined variation in lipid concentrations. This study investigated the association of two single nucleotide polymorphisms (SNPs) at *LPL*, seven tagging SNPs at the *APOE* gene, and a common *APOE* haplotype (two SNPs) with blood lipids, and examined the interaction of these SNPs with dietary factors.

**Methods:**

The population studied for this investigation included 660 individuals from the Prevention of Cancer by Intervention with Selenium (PRECISE) study who supplied baseline data. The findings of the PRECISE study were further replicated using 1238 individuals from the Caerphilly Prospective cohort (CaPS). Dietary intake was assessed using a validated food-frequency questionnaire (FFQ) in PRECISE and a validated semi-quantitative FFQ in the CaPS. Interaction analyses were performed by including the interaction term in the linear regression model adjusted for age, body mass index, sex and country.

**Results:**

There was no association between dietary factors and blood lipids after Bonferroni correction and adjustment for confounding factors in either cohort. In the PRECISE study, after correction for multiple testing, there was a statistically significant association of the *APOE* haplotype (rs7412 and rs429358; E2, E3, and E4) and *APOE* tagSNP rs445925 with total cholesterol (*P* = 4 × 10^− 4^ and *P* = 0.003, respectively). Carriers of the E2 allele had lower total cholesterol concentration (5.54 ± 0.97 mmol/L) than those with the E3 (5.98 ± 1.05 mmol/L) (*P* = 0.001) and E4 (6.09 ± 1.06 mmol/L) (*P* = 2 × 10^− 4^) alleles. The association of *APOE* haplotype (E2, E3, and E4) and *APOE* SNP rs445925 with total cholesterol (P = 2 × 10^− 6^ and *P* = 3 × 10^− 4^, respectively) was further replicated in the CaPS. Additionally, significant association was found between *APOE* haplotype and *APOE* SNP rs445925 with low density lipoprotein cholesterol in CaPS (P = 4 × 10^− 4^ and P = 0.001, respectively). After Bonferroni correction, none of the cohorts showed a statistically significant SNP-diet interaction on lipid outcomes.

**Conclusion:**

In summary, our findings from the two cohorts confirm that genetic variations at the *APOE* locus influence plasma total cholesterol concentrations, however, the gene-diet interactions on lipids require further investigation in larger cohorts.

**Electronic supplementary material:**

The online version of this article (10.1186/s12944-018-0744-2) contains supplementary material, which is available to authorized users.

## Background

Cardiovascular diseases (CVD) are common multifactorial conditions characterized by dyslipidaemia, type 2 diabetes and hypertension [1, 2]. Elevated triacylglycerol (TAG) and reduced high density lipoprotein cholesterol (HDL-C) concentrations are associated with an increased risk of developing CVD [3–5]. Furthermore, several studies have reported that certain genetic variants influence susceptibility to altered circulating lipid concentrations, leading to an increased risk of CVD events [6–8]. Genetic variations have been shown to be associated with lipid outcomes, while dietary factors appear to modulate the effect of such genes on lipid concentrations [9, 10]. Previous studies have shown that single nucleotide polymorphisms (SNPs) of the apolipoprotein E (*APOE*) [6, 11] and lipoprotein lipase (*LPL*) [12–14] genes contribute to significant variation in lipid concentrations.

The APOE protein plays a key role in the transport and metabolism of cholesterol and TAG containing particles by serving as a receptor-binding ligand that mediates the clearance of dietary derived chylomicrons, and hepatically derived very low density lipoprotein (VLDL) and their remnants from the circulation [6]. The three most recognized alleles of the *APOE* gene are E2, E3 and E4, with carriage of E4 associated with CVD risk factors and increased low density lipoprotein cholesterol (LDL-C) concentrations [11, 15, 16], and hence increased CVD risk [17, 18].

Genetic variations in the *LPL* gene have been reported to be involved with lipid metabolism and partly explain the phenotypic variation in blood lipid levels [19]. LPL is a lipolytic enzyme that catalyses hydrolysis of TAG in all of the major classes of TAG-rich lipoproteins [20]. High enzyme activity is associated with favourable lipid levels, including relatively low TAG concentrations [21]. The two most widely studied *LPL* SNPs, rs328 (S447X) and rs320 (HindIII) [22, 23]. The ‘G’ minor alleles of both the SNPs, rs328 and rs320, are associated with decreased TAG concentrations and increased HDL-C concentrations, whereas the opposite association was found for the ‘C’ allele and ‘T’ allele respectively [24–26].

Data from several studies supports the role of genetic factors in lipid metabolism [27]; however, only a few studies have examined the effects of lifestyle factors such as diet on the association of polymorphisms with lipid-related outcomes [10, 28, 29]. Therefore, the present study aimed to investigate the effect of seven *APOE* tagSNPs (rs405509, rs769450, rs439401, rs445925, rs405697, rs1160985, and rs1064725), one *APOE* haplotype (rs7412 and rs429358), and two commonly studied *LPL* SNPs (rs328 and rs320) on blood lipid profile in 660 participants (baseline data) from the Prevention of Cancer by Intervention with Selenium (PRECISE) study. As diet type and intake is also known to modify lipid levels [30–32], the potential impact of the interaction between these SNPs and dietary factors on lipid levels was also investigated. To confirm the findings, the Caerphilly Prospective Study (CaPS; *n* = 1238) was used as a replication cohort.

## Methods

### PRECISE cohort

#### Participants and methods

Baseline data of 660 individuals from the PRECISE study, conducted in two populations [UK (*n* = 468) and Denmark (*n* = 192)] were used for the analysis [33, 34]. Briefly, study participants were selected from four general practices (study centres) in various areas of the UK that were affiliated with the Medical Research Council General Practice Research Framework (MRC GPRF). Between June 2000 and July 2001, research nurses recruited similar numbers of men and women from each of three age groups: 60–64, 65–69 and 70–74 years. The Danish participants were men and women recruited from the same three age groups from the County of Funen in Denmark.

The UK study obtained approval from the appropriate UK Local Research Ethics Committees [South Tees (ref: 99/69), Worcestershire Health Authority (ref: LREC 74/99), Norwich District (ref: LREC 99/ 141), Great Yarmouth and Waveney (under reciprocal arrangements with Norwich District LREC)], and the participants provided written informed consent. The regional Danish Data Protection Agency and Scientific Ethical Committees of Vejle and Funen counties approved the Danish study (Journal number. 19980186).

#### Dietary information

Information about each participant’s usual dietary intake was obtained using validated EPIC food frequency questionnaires (FFQ) [35]. Total energy intake and macronutrient composition were analysed using the FETA software program [36].

#### Anthropometric measurements and biochemical analysis

Body mass index (BMI) was calculated as body weight in kilograms divided by height in square metres (kg/m^2^). Participants provided non-fasting blood samples for biochemical analysis and these samples were stored at − 80 °C. Total cholesterol and HDL-C concentrations in lithium-heparin plasma were measured using an Architect c16000 analyser (Abbott) with dedicated reagents. Measurements were performed by enzymatic colorimetric analysis. Traceability for total cholesterol and HDL-C was ensured through participation in the National Reference System for Cholesterol (NRS/CHOL), as established by the Clinical and Laboratory Standards Institute, with isotope dilution-MS used as the reference method, and reference material taken from the National Institute of Standard and Technology. Evidence of equivalence in the analytical performance of the cholesterol-oxidase assays performed in the UK and Denmark from a comparison of total cholesterol on forty-four serum samples which produced a limit of variation of 2% [33].

#### SNP selection

The *APOE* gene is located on chromosome 19q13.32. It comprises four exons, which are transcribed into the *APOE* mRNA which is 1180 nucleotides long. The seven tagSNPs for the *APOE* gene were chosen based on International HapMap Phase II collected from individuals of Northern and Western European ancestry (CEU) (HapMap Data release 27 Phase 2 + 3, Feb 09, NCBI B36 assembly, dbSNP b126). The Haploview software V3.3 (http://www.broadinstitute.org/haploview/haploview-downloads) was used to assess the linkage disequilibrium between SNPs. Tagger software was used to select tagSNPs with the ‘pairwise tagging only’ option. Two criteria were used to filter the SNPs included in the analysis, minor allele frequency ≥ 5% and Hardy–Weinberg equilibrium *P*-value > 0.01. In total, seven tagSNPs [rs405509 (G > T), rs1160985 (C > T), rs769450 (G > A), rs439401 (C > T), rs445925 (G > A), rs405697 (G > A), and rs1064725 (T > G)] representing the entire common genetic variations across the *APOE* gene were selected for the study. The *APOE* haplotype/SNPs [6, 11, 37–44] and *LPL* [12, 13] SNPs were chosen based on their previous association with various lipid outcomes.

#### DNA isolation and genotyping

The genotyping for the selected SNPs using a KASP assay with a competitive allele-specific PCR assay® was performed on DNA samples by LGC Genomics (Hoddesdon, Herts, UK). The eleven SNPs were in Hardy Weinberg Equilibrium (HWE) (*P* > 0.05 for all comparisons) (Additional file 1: Table S1).

### Caerphilly prospective study (CaPS)

#### Participants and methods

The CaPS was used to replicate the findings from the PRECISE study. The phase 1 (July 1979 to September 1983) recruitment for the CaPS included 2512 men aged 45–59 years who were living in the town of Caerphilly and five of its adjacent villages in the UK; these participants were followed up at regular intervals [45, 46]. The follow-up data collection included periods from 1984 to1988 (phase 2), from 1989 to 1993 (phase 3), from 1993 to 1997 (phase 4), and from 2002 to 2005 (phase 5). For the current study, the data analysed were taken from phase 3 (*n* = 1238), which had the maximum number of samples and variables appropriate to this analysis (total cholesterol and dietary information), and from phase 5 (*n* = 529) (HDL-C and LDL-C). Ethical approval was obtained from the South Wales Research Ethics Committee D, and each subject provided written informed consent.

#### Dietary information

Participants completed validated semi-quantitative FFQ in phase 3 [47, 48]. The FFQ included 50 typical food items in the British diet in order to estimate the mean daily energy intake and macronutrients and micronutrients consumption.

#### Anthropometric measurements and biochemical analysis

Height and weight was recorded in order to calculate the BMI. Height was measured on a stadiometer and weight was measured on a beam balance. Plasm prepared from blood samples taken after an overnight fast were transported at 4 °C to the laboratories on the day of venepuncture. Total cholesterol and HDL-C, LDL-C concentrations were measured using enzymatic procedures [49]. and the LDL-C levels were calculated using the Friedewald Formula [50].

#### DNA isolation and genotyping

DNA was extracted from blood samples collected during the period 1992–1994. SNP information was obtained from the Illumina Cardio Metabochip, which includes data on 200,000 SNPs from regions previously identified for associations with risk factors for cardiometabolic disease [51]. Imputation was conducted against the 1000-genomes reference panel, providing information on approximately two million typed or imputed SNPs. Duplicate samples were genotyped to compute the error rate. Quality control on genotyped samples has been previously reported [52] and the SNPs had a call rate of > 98%. The SNPs were in HWE (*P* > 0.05) (Additional file 1: Table S1).

#### Statistical analysis

Statistical analysis was performed using the SPSS software package, version 22.0. The data were presented as mean ± standard deviation (SD) in Tables 1 and 3 and beta regression coefficients and standard error (SE) were presented in Tables 2, 4, and 5. Independent t-test was used to compare means between men and women at baseline in the PRECISE cohort (Table 1). Univariate linear regression analysis was applied to test for association of the SNPs with total cholesterol and HDL-C, controlling for age, sex, BMI and country. SNP-diet interactions on total cholesterol and HDL-C were investigated using a univariate general linear model. In this model, total cholesterol and HDL-C were the dependent variables, SNPs were fixed factors, and dietary factors (fat energy %, protein energy %, carbohydrate energy %), sex, age BMI, and country were covariates. The dominant model was applied for all SNPs with minor allele frequency ≤ 0.3 and the additive model applied for SNPs with minor allele frequency ≥ 0.4. For analytical purposes, the six *APOE* genotype groups (E2/E2, E2/E3, E3/E3, E3/E4, E4/E4, and E2/E4) were classified into three groups. The E3/E3 genotype was classified as a group as it occurs at high frequency in the population (wild type). The E2/E2 and E2/E3 genotypes were combined and presented as E2 carriers. The E3/E4 and E4/E4 genotypes were also combined, and presented as E4 carriers [29]. Previous studies have shown that the impact of the E2 allele on serum lipids is greater than that of the E4 allele [17], therefore, the E2/E4 genotype was excluded from the analysis. The Bonferroni correction was applied separately for association and interaction analyses. For association between phenotypic and dietary factors, the Bonferroni-corrected *P* value was 0.008 (2 lipid outcomes* 3 dietary factors) for the PRECISE study and P value was 0.01 for CaPS (total cholesterol was the only variable available). For association between SNPs and lipids (PRECISE study), the Bonferroni corrected P value was 0.003 (10 SNPs*2 lipid outcomes = 20 tests). For interactions (PRECISE study), the Bonferroni corrected P value was 0.001 (10 SNPs*2 lipid outcomes*3 dietary factors = 60 tests). In the replication analysis (CaPS cohort), the Bonferroni corrected P value for association was 0.002 (10 SNPs*3 lipid outcomes = 30 tests), while for interactions it was 0.001 (10 SNPs*1 lipid outcome* 3 dietary factors = 30 tests).

**Table 1:** Baseline characteristics of the PRECISE and Caerphilly Prospective study participants

	PRECISE study			Caerphilly Prospective study (CaPS)
Characteristics	Men (*N* = 248 UK, 95 Danish)	Women (*N* = 220 UK, 97 Danish)	*P* value	Men (*N* = 1238)
Age (years)	67 ± 4	67 ± 4	0.12	62 ± 4
Body mass index (kg/m^2^)	27.2 ± 4.9	27.3 ± 4.9	0.82	26.8 ± 3.7
Total Cholesterol (mmol/L)	5.6 ± 0.9	6.2 ± 1.1	2.31 × 10^−10^	6.1 ± 1.1
High density lipoprotein cholesterol (mmol/L)^a^	1.5 ± 0.3	1.7 ± 0.4	2.71 × 10^− 16^	1.3 ± 0.3
Protein intake (total energy %)	17.6 ± 3.7	18.8 ± 3.7	5X10^− 5^	14.9 ± 2.7
Carbohydrate intake (total energy %)	42.8 ± 13.3	48.2 ± 8.7	1.42 × 10^− 9^	48.4 ± 7.5
Fat intake (total energy %)	35.3 ± 7.1	33.9 ± 6.9	0.01	36.5 ± 6.9
Total energy intake (kcal)	2256 ± 658	1992 ± 613	2.63 × 10^−7^	1964 ± 625
Total energy intake (MJ)	9.4 ± 2.7	8.3 ± 2.6	2.63 × 10^−7^	8.2 ± 2.6

Data shown are represented as means ± SD, wherever appropriate. *P* values are for the differences in the means between men and women. *P* values were calculated by using independent t-test

^a^For CaPS, HDL-C levels were obtained from phase 5 while all other variables were obtained from phase 3

**Table 2:** Association between dietary factors and lipids in PRECISE and Caerphilly Prospective studies

PRECISE study		
Association between dietary factors and total cholesterol		
Fat total energy % intake Beta (± S.E), P_association_	Protein total energy % intake Beta (± S.E), P_association_	Carbohydrate total energy % intake Beta (± S.E), P_association_
0.01 (0.01) 0.47	−0.01 (0.01) 0.13	−0.004 (0.01) 0.40
Association between three dietary factors and HDL-C high density lipoprotein		
Fat total energy % intake	Protein total energy % intake	Carbohydrate total energy % intake
−0.002 (0.002) 0.29	−0.002 (0.004) 0.59	− 0.004 (0.002) 0.02
Caerphilly Prospective study		
Association between three dietary factors and total cholesterol		
Fat total energy % intake Beta (± S.E), P_association_	Protein total energy % intake Beta (± S.E), P_association_	Carbohydrate total energy % intake Beta (± S.E), P_association_
0.01 (0.004) 0.06	−0.01 (0.01) 0.26	−0.01 (0.004) 0.17

HDL-C, high density lipoprotein cholesterol

*P* values were obtained using linear regression adjusted for age, sex, body mass index and country

## Results

### Participant characteristics

The general characteristics of the participants by sex are presented in Table 1. In the PRECISE study, women were found to have significantly higher total cholesterol and HDL-C concentrations than men (*P* = 2.31 × 10^− 10^ and *P* = 2.71 × 10^− 16^, respectively). The consumption of carbohydrates (*P* = 1.42 × 10^− 9^) and protein (energy %) (*P* = 5 × 10^− 5^) were higher in women than in men, whereas the consumption of fat (energy %) and total energy intake were lower in women than in men (*P* = 0.01). Characteristics of the individuals from CaPS are given in Table 1. Elevated total cholesterol levels were observed among men at phase 3. Dietary-pattern data showed higher consumption of energy from total fat.

### Association between dietary factors and blood lipids

In both the PRECISE and CaPS, there was no association between the dietary factors and total cholesterol or high-density lipoprotein after Bonferroni correction and adjustment for confounding factors (Table 2).

### Genotypes and serum lipid levels in the PRECISE study

As shown in Table 3, of the seven tagSNPs at *APOE*, tagSNP rs445925 was significantly associated with total cholesterol (*P* = 0.003) after correction for multiple testing. The ‘A’ allele carriers (5.65 ± 0.98 mmol/L) had 5% lower levels of total cholesterol than GG homozygotes (5.99 ± 1.06 mmol/L).

**Table 3:** Association of *APOE* and *LPL* SNPs with HDL-C and total cholesterol levels in the PRECISE and Caerphilly studies

SNP	MAF	HDL-C (mmol/L)	Total Cholesterol (mmol/L)	LDL-C ^a^ (mmol/L)
PRECISE				
*LPL*				
rs320	0.26			
TT		1.6 ± 0.3	5.9 ± 1.1	
T/G		1.7 ± 0.4	5.8 ± 1.0	
*P* value		0.02	0.19	
rs328	0.10			
CC		1.6 ± 0.3	5.9 ± 1.1	
C/G		1.7 ± 0.4	5.7 ± 0.9	
*P* value		0.04	0.06	
*APOE*				
rs405509	0.47			
GG		1.7 ± 0.4	5.8 ± 1.1	
GT		1.5 ± 0.3	5.8 ± 1.1	
TT		1.6 ± 0.3	6.1 ± 1.0	
*P* value		0.07	0.23	
rs769450	0.39			
GG		1.6 ± 0.3	5.9 ± 1.1	
A allele		1.6 ± 0.4	5.9 ± 1.1	
*P* value		0.72	0.97	
rs439401	0.33			
CC		1.6 ± 0.4	5.9 ± 1.1	
T allele		1.6 ± 0.3	5.9 ± 1.1	
*P* value		0.43	0.51	
rs445925	0.11			
GG		1.6 ± 0.3	5.9 ± 1.1	
A allele		1.7 ± 0.4	5.6 ± 0.9	
*P* value		0.25	0.003	
rs405697	0.25			
GG		1.6 ± 0.4	5.9 ± 1.1	
A allele		1.6 ± 0.3	5.9 ± 1.0	
*P* value		0.71	0.96	
rs1160985	0.43			
CC		1.6 ± 0.3	5.9 ± 1.1	
CT		1.6 ± 0.4	5.8 ± 1.0	
TT		1.7 ± 0.4	5.9 ± 1.1	
*P* value		0.12	0.44	
rs1064725	0.04			
TT		1.6 ± 0.4	5.9 ± 1.0	
G allele		1.7 ± 0.3	6.1 ± 1.2	
*P* value		0.17	0.38	
(rs7412- rs429358) E2, E3, and E4				
E3		1.6 ± 0.3	5.9 ± 1.1	
E4		1.5 ± 0.3	6.1 ± 1.1	
E2		1.7 ± 0.4	5.5 ± 0.9	
*P* value		0.09	4X10^−4^	
Caerphilly				
*LPL*				
rs320	0.26			
TT		1.3 ± 0.3	6.1 ± 1.1	2.7 ± 0.8
T/G		1.4 ± 0.3	6.2 ± 1.2	2.8 ± 0.8
*P* value		0.05	0.55	0.05
rs328	0.10			
CC		1.3 ± 0.3	6.1 ± 1.1	2.7 ± 0.8
C/G		1.3 ± 0.3	6.1 ± 1.1	2.9 ± 0.9
*P* value		0.63	0.71	0.05
*APOE*				
rs405509	0.46			
GG		1.4 ± 0.3	6.0 ± 1.1	2.7 ± 0.9
GT		1.3 ± 0.3	6.2 ± 1.1	2.8 ± 0.8
TT		1.3 ± 0.3	6.3 ± 1.1	2.9 ± 0.9
*P* value		0.16	0.02	0.29
rs769450	0.41			
GG		1.3 ± 0.2	6.1 ± 1.2	2.8 ± 0.9
A allele		1.4 ± 0.3	6.2 ± 1.1	2.8 ± 0.8
*P* value		0.10	0.41	0.82
rs439401	0.35			
CC		1.4 ± 0.3	6.2 ± 1.1	2.8 ± 0.9
T allele		1.3 ± 0.3	6.1 ± 1.1	2.7 ± 0.8
*P* value		0.72	0.42	0.32
rs445925	0.11			
GG		1.3 ± 0.3	6.2 ± 1.1	2.8 ± 0.8
A allele		1.3 ± 0.3	5.9 ± 1.2	2.5 ± 0.9
*P* value		0.99	3X10^−4^	0.001
rs405697	0.26			
GG		1.4 ± 0.4	6.1 ± 1.1	2.8 ± 0.9
A allele		1.3 ± 0.3	6.1 ± 1.1	2.8 ± 0.8
*P* value		0.30	0.88	0.9
rs1160985	0.45			
CC		1.34 ± 0.29	6.2 ± 1.1	2.8 ± 0.9
CT		1.35 ± 0.35	6.2 ± 1.2	2.7 ± 0.8
TT		1.37 ± 0.40	6.1 ± 1.0	2.8 ± 0.8
*P* value		0.61	0.30	0.73
rs1064725	0.01			
TT		1.3 ± 0.3	6.2 ± 1.1	2.8 ± 0.8
G allele		1.4 ± 0.3	6.1 ± 1.1	2.8 ± 0.7
*P* value		0.18	0.60	0.68
(rs7412- rs429358) E2, E3, and E4				
E3		1.4 ± 0.4	6.2 ± 1.1	2.8 ± 0.8
E4		1.4 ± 0.3	6.4 ± 1.1	3.0 ± 0.9
E2		1.3 ± 0.3	5.8 ± 1.3	2.4 ± 0.8
*P* value		0.95	2X10^−6^	4X10^−4^

Values are given as mean ± SD. *P* values for differences between genotypes were obtained using linear regression model adjusted for age, sex, body mass index, and country

Bonferroni corrected *P* value < 0.003 was considered statistically significant

*MAF* minor allele frequency, *HDL-C* high density lipoprotein cholesterol, *LDL-C* low density lipoprotein cholesterol

^a^ LDL-C values available only in Caerphilly prospective study

The levels of HDL-C were significantly different among the *LPL* SNP genotypes, rs328 (*P* = 0.04) and rs320 (*P* = 0.02), where the carriers of the ‘G’ minor allele of both SNPs had higher levels of HDL-C (1.68 ± 0.41 mmol/L for rs328 and 1.66 ± 0.40 mmol/L for rs320) than CC homozygotes (rs328) and TT homozygotes (rs320) (1.61 ± 0.38 and 1.60 ± 0.39 mmol/L) respectively. However, these associations were not statistically significant after Bonferroni correction.

### APOE haplotype and serum lipid levels in the PRECISE study

**Fig. 1:**
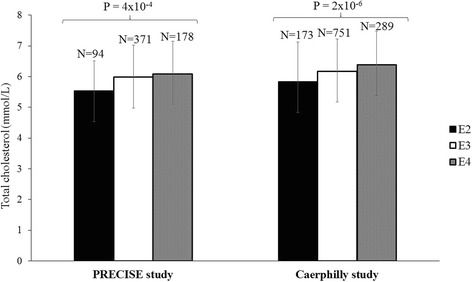
Association of *APOE* haplotypes (E2, E3, and E4) with total cholesterol concentrations in the Prevention of Cancer by Intervention with Selenium (PRECISE) study and Caerphilly Prospective study (CaPS). E2 allele carriers have significantly lower levels of total cholesterol than E3 (*P* = 0.001 and *P* = 4 × 10^− 4^ in the PRECISE and CaPS, respectively) and E4 (*P* = 2 × 10^− 4^ and *P* = 3 × 10^− 6^ in the PRECISE and CaPS, respectively) allele carriers

The effects of *APOE* haplotypes (E2, E3, and E4) on serum lipids are shown in Table 3. These haplotypes (E2, E3, and E4) were significantly associated with total cholesterol (*P* = 4 × 10^− 4^) after correction for multiple testing. The carriers of the E2 allele (5.54 ± 0.97 mmol/L) had lower total cholesterol concentrations than the carriers of the E3 (*P* = 0.001) (5.98 ± 1.05 mmol/L) and E4 alleles (6.09 ± 1.06 mmol/L) (*P* = 2 × 10^− 4^) (Fig. 1).

### Interactions between genotypes and dietary factors on serum lipid in the PRECISE study

None of the dietary factors significantly interacted with the *APOE* SNPs, haplotypes and *LPL* SNPs with plasma lipids after correction for multiple testing (*P* > 0.001) (Table 4).

**Table 4:** Interaction between *APOE* and *LPL* SNPs and dietary factors on HDL-C and total cholesterol in the PRECISE study

Interaction between rs320 at LPL*dietary factors on HDL-C		
Interaction between SNP rs320* fat energy % intake	Interaction between SNP rs320* protein energy % intake	Interaction between SNP rs320* carbohydrate energy % intake
0.003 (0.004) 0.46	0.002 (0.01) 0.76	−0.0004 (0.002) 0.87
Interaction between rs320 at *LPL* *dietary factors on Total Cholesterol		
Interaction between SNP rs320* fat energy % intake	Interaction between SNP rs320* protein energy % intake	Interaction between SNP rs320* carbohydrate energy % intake
0.01(0.01) 0.27	−0.03 (0.02) 0.13	−0.01 (0.01) 0.06
Interaction between rs328 at *LPL* *dietary factors on HDL-C		
Interaction between SNP rs328* fat energy % intake	Interaction between SNP rs328* protein energy % intake	Interaction between SNP rs328* carbohydrate energy % intake
0.01 (0.01) 0.09	−0.001 (0.01) 0.89	0.001 (0.003) 0.63
Interaction between rs328 at *LPL* *dietary factors on Total Cholesterol		
Interaction between SNP rs328* fat energy % intake	Interaction between SNP rs328* protein energy % intake	Interaction between SNP rs328* carbohydrate energy % intake
−0.002 (0.02) 0.88	0.003 (0.03) 0.90	−0.01 (0.01) 0.55
Interaction between rs405509 at APOE*dietary factors on HDL-C		
Interaction between SNP rs405509* fat energy % intake	Interaction between SNP rs405509* protein energy % intake	Interaction between SNP rs405509* carbohydrate energy % intake
0.01 (0.01) 0.11	−0.001 (0.01) 0.75	−0.01 (0.003) 0.09
Interaction between rs405509 at *APOE* *dietary factors on Total Cholesterol		
Interaction between SNP rs405509* fat energy % intake	Interaction between SNP rs405509* protein energy % intake	Interaction between SNP rs405509* carbohydrate energy % intake
0.02 (0.02) 0.39	−0.04 (0.03) 0.26	−0.01 (0.01) 0.59
Interaction between rs769450 at *APOE* *dietary factors on HDL-C		
Interaction between SNP rs769450* fat energy % intake	Interaction between SNP rs769450* protein energy % intake	Interaction between SNP rs769450* carbohydrate energy % intake
−0.001 (0.004) 0.88	0.001 (0.01) 0.88	0.003 (0.003) 0.19
Interaction between rs769450 at *APOE* *dietary factors on Total Cholesterol		
Interaction between SNP rs769450* fat energy % intake	Interaction between SNP rs769450* protein energy % intake	Interaction between SNP rs769450* carbohydrate energy % intake
−0.001 (0.01) 0.94	0.01 (0.02) 0.63	0.01 (0.01) 0.51
Interaction between rs439401 at *APOE* *dietary factors on HDL-C		
Interaction between SNP rs439401* fat energy % intake	Interaction between SNP rs439401* protein energy % intake	Interaction between SNP rs439401* carbohydrate energy % intake
0.01 (0.004) 0.11	0.01 (0.01) 0.39	−0.001 (0.003) 0.64
Interaction between rs439401 at *APOE* *dietary factors on Total Cholesterol		
Interaction between SNP rs439401* fat energy % intake	Interaction between SNP rs439401* protein energy % intake	Interaction between SNP rs439401* carbohydrate energy % intake
0.003 (0.01) 0.79	−0.02 (0.02) 0.37	− 0.001 (0.01) 0.89
Interaction between rs445925 at *APOE* *dietary factors on HDL-C		
Interaction between SNP rs445925* fat energy % intake	Interaction between SNP rs445925* protein energy % intake	Interaction between SNP rs445925* carbohydrate energy % intake
−0.003 (0.01) 0.53	0.01 (0.01) 0.52	0.0003 (0.003) 0.93
Interaction between rs445925 at *APOE* *dietary factors on Total Cholesterol		
Interaction between SNP rs445925* fat energy % intake	Interaction between SNP rs445925* protein energy % intake	Interaction between SNP rs445925* carbohydrate energy % intake
−0.03 (0.01) 0.05	0.01 (0.03) 0.66	0.01 (0.01) 0.36
Interaction between rs405697 at *APOE* *dietary factors on HDL-C		
Interaction between SNP rs405697* fat energy % intake	Interaction between SNP rs405697* protein energy % intake	Interaction between SNP rs405697* carbohydrate energy % intake
0.01(0.004) 0.06	−0.002 (0.01) 0.80	−0.004 (0.002) 0.16
Interaction between rs405697 at *APOE* *dietary factors on Total Cholesterol		
Interaction between SNP rs405697* fat energy % intake	Interaction between SNP rs405697* protein energy % intake	Interaction between SNP rs405697* carbohydrate energy % intake
0.01 (0.01) 0.22	−0.03 (0.02) 0.19	−0.003 (0.01) 0.72
Interaction between rs1160985 at *APOE* *dietary factors on HDL-C		
Interaction between SNP rs1160985* fat energy % intake	Interaction between SNP rs1160985* protein energy % intake	Interaction between SNP rs1160985* carbohydrate energy % intake
−0.01 (0.01) 0.08	−0.002 (0.01) 0.97	0.01 (0.004) 0.03
Interaction between rs1160985 at *APOE* *dietary factors on Total Cholesterol		
Interaction between SNP rs1160985* fat energy % intake	Interaction between SNP rs1160985* protein energy % intake	Interaction between SNP rs1160985* carbohydrate energy % intake
−0.01 (0.01) 0.58	0.05 (0.03) 0.28	−0.001 (0.01) 0.19
Interaction between rs1064725 at *APOE* *dietary factors on HDL-C		
Interaction between SNP rs1064725* fat energy % intake	Interaction between SNP rs1064725* protein energy % intake	Interaction between SNP rs1064725* carbohydrate energy % intake
−0.001 (0.01) 0.90	0.004 (0.02) 0.77	−0.002 (0.004) 0.73
Interaction between rs1064725 at *APOE* *dietary factors on Total Cholesterol		
Interaction between SNP rs1064725* fat energy % intake	Interaction between SNP rs1064725* protein energy % intake	Interaction between SNP rs1064725* carbohydrate energy % intake
0.03 (0.03) 0.28	0.02 (0.04) 0.62	−0.01 (0.01) 0.48
Interaction between *APOE* (E2, E3, and E4)*dietary factors on HDL-C		
Interaction between SNP *APOE* (E2, E3, and E4)* fat energy % intake	Interaction between SNP *APOE* (E2, E3, and E4)* protein energy % intake	Interaction between SNP *APOE* (E2, E3, and E4)* carbohydrate energy % intake
−0.01 (0.01) 0.39	0.001 (0.01) 0.99	0.002 (0.003) 0.17
Interaction between *APOE* (E2, E3, and E4)*dietary factors on Total Cholesterol		
Interaction between SNP *APOE* (E2, E3, and E4)* fat energy % intake	Interaction between SNP *APOE* (E2, E3, and E4)* protein energy % intake	Interaction between SNP *APOE* (E2, E3, and E4)* carbohydrate energy % intake
−0.03 (0.02) 0.18	−0.02 (0.04) 0.32	0.01 (0.01) 0.51

Values represented β regression coefficients (± S.E), and P_interaction_. P values were obtained by using a general linear model adjusted for age, sex, body mass index, country and total energy intake, wherever appropriate

Bonferroni corrected *P* value < 0.001 was considered statistically significant

*HDL-C* High density lipoprotein cholesterol

### Replication analysis: Effect of SNPs at APOE and LPL on serum lipids in the CaPS

The associations of *APOE* and *LPL* SNPs with blood lipids in the CaPS are presented in Table 3. The association of *APOE* haplotype (E2, E3, and E4) and *APOE* SNP rs445925 with total cholesterol (P = 2 × 10^− 6^ and *P* = 3 × 10^− 4^, respectively) was replicated (Fig. 1). The ‘A’ allele carriers of *APOE* SNP rs445925 had lower total cholesterol (5.96 ± 1.24 mmol/l) than ‘GG’ genotypes (6.24 ± 1.08 mmol/L). In the *APOE* haplotype analysis, the carriers of the E2 allele had 5% and 14% lower total cholesterol than carriers of the E3 (*P* = 4 × 10^− 4^) and E4 alleles (P = 3 × 10^− 6^), respectively. Additionally, significant association was seen between *APOE* haplotypes (E2, E3, and E4) and *APOE* SNP rs445925and LDL-C (P = 4X10^− 4^, 0.001, respectively).

There was an interaction between fat (% energy) and *APOE* haplotype (E2, E3, and E4) on total cholesterol (*P* = 0.038) in CaPS. However, after correction for multiple testing, all the SNP-diet interactions were consistent with chance variation (Table 5).

**Table 5:** Interaction between *APOE* and *LPL* SNPs and dietary factors on total cholesterol in the CaPS

Interaction between rs320 at *LPL* *dietary factors on Total Cholesterol		
Interaction between SNP rs320* fat energy % intake	Interaction between SNP rs320* protein energy % intake	Interaction between SNP rs320* carbohydrate energy % intake
0.01 (0.01) 0.48	− 0.01 (0.03) 0.57	− 0.004 (0.01) 0.64
Interaction between rs328 at *LPL* *dietary factors on Total Cholesterol		
Interaction between SNP rs328* fat energy % intake	Interaction between SNP rs328* protein energy % intake	Interaction between SNP rs328* carbohydrate energy % intake
− 0.01 (0.01) 0.58	−0.04 (0.03) 0.17	0.01 (0.01) 0.29
Interaction between rs405509 at *APOE* *dietary factors on Total Cholesterol		
Interaction between SNP rs405509* fat energy % intake	Interaction between SNP rs405509* protein energy % intake	Interaction between SNP rs405509* carbohydrate energy % intake
0.03 (0.01) 0.11	−0.04 (0.04) 0.52	−0.02 (0.01) 0.31
Interaction between rs769450 at *APOE* *dietary factors on Total Cholesterol		
Interaction between SNP rs769450* fat energy % intake	Interaction between SNP rs769450* protein energy % intake	Interaction between SNP rs769450* carbohydrate energy % intake
−0.01 (0.01) 0.10	0.05 (0.02) 0.04	0.01 (0.01) 0.42
Interaction between rs439401 at *APOE* *dietary factors on Total Cholesterol		
Interaction between SNP rs439401* fat energy % intake	Interaction between SNP rs439401* protein energy % intake	Interaction between SNP rs439401* carbohydrate energy % intake
−0.003 (0.01) 0.77	−0.01 (0.03) 0.68	0.004 (0.01) 0.65
Interaction between rs445925 at *APOE* *dietary factors on Total Cholesterol		
Interaction between SNP rs445925* fat energy % intake	Interaction between SNP rs445925* protein energy % intake	Interaction between SNP rs445925* carbohydrate energy % intake
−0.0003 (0.01) 0.97	−0.02 (0.03) 0.55	0.002 (0.01) 0.87
Interaction between rs405697 at *APOE* *dietary factors on Total Cholesterol		
Interaction between SNP rs405697* fat energy % intake	Interaction between SNP rs405697* protein energy % intake	Interaction between SNP rs405697* carbohydrate energy % intake
0.01 (0.01) 0.51	−0.03 (0.03) 0.24	−0.002 (0.01) 0.84
Interaction between rs1160985 at *APOE* *dietary factors on Total Cholesterol		
Interaction between SNP rs1160985* fat energy % intake	Interaction between SNP rs1160985* protein energy % intake	Interaction between SNP rs1160985* carbohydrate energy % intake
−0.01 (0.01) 0.13	−0.004 (0.03) 0.19	0.01 (0.01) 0.43
Interaction between rs1064725 at *APOE* *dietary factors on Total Cholesterol		
Interaction between SNP rs1064725* fat energy % intake	Interaction between SNP rs1064725* protein energy % intake	Interaction between SNP rs1064725* carbohydrate energy % intake
−0.01 (0.03) 0.66	0.05 (0.11) 0.62	0.01 (0.03) 0.74
Interaction between *APOE* (E2,E3, and E4)*dietary factors on Total Cholesterol		
Interaction between SNP *APOE* (E2, E3, and E4)* fat energy % intake	Interaction between SNP *APOE* (E2, E3, and E4)* protein energy % intake	Interaction between SNP *APOE* (E2, E3, and E4)* carbohydrate energy % intake
−0.02 (0.02) 0.038	0.02 (0.04) 0.83	0.01 (0.01) 0.08

Values represented β regression coefficients (± S.E), and P_interaction_

P values were obtained by using a general linear model adjusted for age, sex, body mass index, country and total energy intake, wherever appropriate

Bonferroni corrected *P* value < 0.001 was considered statistically significant

## Discussion

Our findings demonstrated significant associations between the *APOE* haplotype (E2, E3, and E4) and *APOE* SNP rs445925 with total plasma cholesterol and LDL-C (only CaPS) concentration, which were further replicated in an independent UK Caucasian cohort. The levels of total cholesterol were significantly lower in carriers of the *APOE* E2 allele and the ‘A’ allele of the SNP rs445925 than carriers of E3, E4 and ‘GG’ genotype of the *APOE* SNP rs445925, respectively. Given that our findings confirm that genetic polymorphisms of *APOE* influence the inter-individual variation in total plasma cholesterol, a marker of dyslipidemia, changes in dietary consumption to reduce disease susceptibility could be implemented for individuals at genetic risk.

The effects of *APOE* polymorphisms on lipid concentrations have previously been investigated in different ethnic groups [11, 53, 54] and studies have shown that the *APOE* gene variants contributed to 7% variability in total cholesterol [55]. The results of the current study were in line with previously reported findings that *APOE* haplotypes (E2, E3, and E4) are associated with serum total cholesterol and LDL-C, with E4 carriers associated with increased concentrations compared with E3/E3 wildtype and particularly E2 carriers [16, 53, 56]. One of the primary roles of APOE is binding the low density lipoprotein receptor (LDLR) and the LDLR-related protein, to facilitate cellular uptake of lipoprotein particles [57]. The three alleles, E2, E3, and E4, differ in their amino-acid sequences, resulting in functional differences in receptors-binding affinity. Amino-acid sequences of the E2 allele have lower binding affinity than those of the E3 and E4 alleles, causing decreased hepatic VLDL and chylomicron remnants clearance, thus reducing the uptake of postprandial lipoprotein particles [57]. Furthermore, it could be postulated that increase in apoE TAG-rich lipoproteins in E4 carriers could possibly increase the affinity to bind LDL-receptors resulting in decreased uptake of LDL and increased circulating plasma cholesterol [58]. E2 carriers also have an impaired conversion of the VLDL particles to LDL-C compared to E4 carriers [59], who have a higher rate of VLDL catabolism [60], which explains in part the lower total cholesterol and LDL-C in E2 allele carriers.

Furthermore, our study highlights an association between *APOE* SNP rs445925, which is one of the selected tagSNPs within the *APOE* gene, and total cholesterol. The SNP rs445925 has not been extensively studied, however, a genome-wide association study showed a significant association between SNP rs445925 and LDL-C levels in 3644 black and white individuals from the US and Europe [61]. In addition, previous genome-wide linkage and association studies have shown linkage disequilibrium (LD) between *APOE* SNPs rs7412 and rs445925 [62] and between ‘A’ allele carriers at SNP rs445925 and E2 haplotype [63], respectively, which could explain in part a similar function in cholesterol synthesis. It is also possible that A’ allele carriers of the SNP rs445925 might exhibit lower conversion of the VLDL particles to LDL-C which could have resulted in the decreased rate of LDL formation and hence lowered the total cholesterol concentrations [63].

Besides genetic associations, our study also identified an interaction of *APOE* haplotypes (E2, E3, and E4) with intake from fat (%) on total cholesterol in the CaPS, where, among those who consumed a low-fat diet (%), individuals carrying the E2 allele had significantly lower total cholesterol concentrations than to E4 allele carriers. However, this interaction was not statistically significant after correction for multiple testing. A previous study has examined the response of *APOE* genotype to fat intake in 45 individuals using a prospective design, where after consumption of a lower-fat-cholesterol diet (34% fat, 265 mg/day) according to modified National Cholesterol Education program there was a significant reduction in total cholesterol by 14%, 9%, and 4% in E4/E4, E3/E4, and E3/E3 genotypes, respectively [64]. Another study showed that the response to a diet high in cholesterol increases total cholesterol in E3 and E4 compared to E2 allele carries in a study comprising 29 healthy men [65]. By contrast, a cross sectional study in European Caucasians (*n* = 996) reported that E2 allele carriers had lower total cholesterol levels, but there were no reported between interactions between saturated fatty acids and total cholesterol [66]. Given that the previous studies have given inconsistent results and have used various types of fatty acids, replication of our gene-diet interaction finding in a large well-designed randomized controlled trial is highly warranted.

Previous studies have shown that the minor allele of *LPL* SNP rs328 enhance lipolytic activity [12]. Increased activity of LPL results in enhance clearance of TAG from the circulation, and associated with higher HDL-C concentrations [67]. The *LPL* SNP rs320 (HindIII) is in LD with rs328 (S447X) and they have been shown to have similar effects on HDL-C, where minor allele was reported to increase HDL-C [24, 68]. In our study, in accordance with findings from other studies, there were associations between *LPL* SNPs, rs320 and rs328, and HDL-C concentrations, where common homozygotes of both SNPs had lower HDL-C [22–24, 26]. However, in our study, these associations were no longer statistically significant after Bonferroni correction. Furthermore, there were no significant *LPL* SNP-diet interactions with HDL-C or total cholesterol concentrations in either cohort. To date, there has only been one study that has shown an interaction between *LPL* rs328 and total fat intake on HDL-C in 8764 individuals from the US population, where high fat intake associated with increase HDL-C in CC homozygotes and CG heterozygotes carriers [28]. One of the main reasons we did not identify a significant interaction may be our small sample size; however, we cannot rule out an effect of differences in dietary fat sources between European and the US population.

The present study has some limitations. Importantly, some lipid-related outcomes, such as LDL-C and TAG concentrations, were not measured in the PRECISE study. The PRECISE study was also conducted in two populations, a UK cohort and a Danish cohort, which used different food frequency questionnaires and this might have introduced measurement bias, even though the current results were adjusted for country in the regression analysis to avoid confounding. Another possible limitation is the use of a cross-sectional design (in both studies) to investigate genetic effects at a single point in time, whereas a longitudinal analysis design would have captured the genetic effects on lipid outcomes over a specific time period. The effect-size of the minor allele of some of the studied SNPs was relatively small, and hence a large sample size is required to detect reliably detect any interaction between SNPs and dietary factors. Despite the fact that this study was not adequately powered to detect such an interaction, it was sufficiently powered to detect the main effects (i.e., associations). Significant gene-diet interactions were identified, however these did not reach the Bonferroni-corrected *P* value (*P* = 0.001) and hence need to be confirmed in larger cohorts. This study is strengthened by the fact that it is the first study to investigate the role of tagSNPs at the *APOE* gene in relation to dietary factors and lipid outcomes. The fact that genetic associations from the PRECISE study were replicated in another Caucasian cohort (CaPS) confirms the validity of our findings. Additionally, CaPS was based on a cohort with a very high response rate, and is therefore closely representative of the general population.

## Conclusion

Our study, carried out in two Caucasian populations, confirmed that genetic variations at the *APOE* gene locus influence plasma lipid concentrations. Thus, our results suggest that *APOE* gene variants affect risk of dyslipidemia in individuals who carry the E4 risk allele and GG genotype at SNP rs445925. Future studies with a larger sample size examining tagSNPs at *APOE*, particularly prospectively genotyped dietary intervention studies are required to confirm the gene-diet interactions identified in our study.

## Additional file

Additional file 1: **Table S1**. Genotype distribution of SNPs at *LPL* and *APOE* genes and Hardy Weinberg Equilibrium *P* values. (DOCX 18 kb)

## Abbreviations

*APOE*: Aspolipoprotein E
CaPS: Caerphilly Prospective cohort
CVD: Cardiovascular disease
FFQ: Food frequency questionnaire
HDL-C: High density lipoprotein cholesterol
HWE: Hardy Weinberg Equilibrium
LD: Linkage disequilibrium
LDL-C: Low density lipoprotein cholesterol
*LPL*: Lipoprotein lipase
PRECISE: Prevention of Cancer by Intervention with Selenium
SNPs: Single nuclide polymorphisms
TAG: Triacylglycerol
VLDL: Very low density lipoprotein
