# Supplementary material for: Association of apolipoprotein E gene polymorphisms with blood lipids and their interaction with dietary factors
Source: Lipids Health Dis. 2018 Apr 30;17:98. doi: 10.1186/s12944-018-0744-2 (PMC5928585; doi:10.1186/s12944-018-0744-2)
Supplement: Supplementary file 1 — Table S1. Genotype distribution of SNPs at LPL and APOE genes and Hardy Weinberg Equilibrium P values. (DOCX 18 kb) [file 12944_2018_744_MOESM1_ESM.docx]

**Table S1: Genotype distribution of SNPs at *LPL* and *APOE* genes and Hardy Weinberg Equilibrium P values**

| **SNP** | **Common homozygous N (%)** | **Heterozygous N (%)** | **Rare homozygous N (%)** | **Chi square** | **HWE P value** |
| --- | --- | --- | --- | --- | --- |
| PRECISE | | | | | |
| rs320 | 354 (0.53) | 271 (0.40) | 39 (0.05) | 1.8 | 0.17 |
| rs328 | 522 (0.80) | 127 (0.19) | 3 (0.005) | 2.6 | 0.11 |
| rs405509 | 183 (27.6%) | 330 (49.8%) | 149 (22.5%) | 0.0001 | 0.99 |
| rs769450 | 228 (34.5%) | 339 (51.4%) | 92 (13.8%) | 3.66 | 0.06 |
| rs439401 | 291 (44.2%) | 290 (44.1%) | 76 (11.5%) | 0.08 | 0.77 |
| rs445925 | 506 (77.3%) | 142 (21.7%) | 6 (0.9%) | 1.33 | 0.24 |
| rs405697 | 365 (54.9%) | 257 (38.7%) | 42 (6.3%) | 0.13 | 0.71 |
| rs1160985 | 200 (30.2%) | 344 (52%) | 117 (17.7%) | 2.18 | 0.13 |
| rs1064725 | 606 (91.4%) | 56 (8.4%) | 1 (0.1%) | 0.06 | 0.81 |
|  |  |  |  |  |  |
| Caerphilly Prospective study | | | | | |
| rs320 | 721 (0.53) | 536 (0.39) | 86 (0.06) | 1.05 | 0.31 |
| rs328 | 1068 (0.79) | 266 (0.19) | 9 (0.006) | 3.01 | 0.08 |
| rs405509 | 381 (0.28) | 675 (0.50) | 287 (0.21) | 0.13 | 0.71 |
| rs769450 | 452(0.33) | 672(0.50) | 219(0.16) | 1.35 | 0.24 |
| rs439401 | 560 (0.41) | 615 (0.45) | 168 (0.12) | 0.0018 | 0.96 |
| rs445925 | 1056 (0.78) | 271 (0.20) | 16 (0.01) | 0.08 | 0.77 |
| rs405697 | 728 (0.54) | 513 (0.38) | 102 (0.07) | 0.77 | 0.38 |
| rs1160985 | 394 (0.29) | 688 (0.51) | 261 (0.19) | 1.61 | 0.20 |
| rs1064725 | 1076 (0.96) | 35 (0.03) | 0 | 0.28 | 0.59 |

HWE; Hardy Weinberg Equilibrium
